# Practical considerations for estimating clinical trial accrual periods: application to a multi-center effectiveness study

**DOI:** 10.1186/1471-2288-5-11

**Published:** 2005-03-30

**Authors:** Rickey E Carter, Susan C Sonne, Kathleen T Brady

**Affiliations:** 1 Department of Biostatistics, Bioinformatics and Epidemiology, Medical University of South Carolina, Charleston, SC, USA; 2 Department of Psychiatry and Behavioral Sciences, Medical University of South Carolina, Charleston, SC, USA

## Abstract

**Background:**

Adequate participant recruitment is vital to the conduct of a clinical trial. Projected recruitment rates are often over-estimated, and the time to recruit the target population (accrual period) is often under-estimated.

**Methods:**

This report illustrates three approaches to estimating the accrual period and applies the methods to a multi-center, randomized, placebo controlled trial undergoing development.

**Results:**

Incorporating known sources of accrual variation can yield a more justified estimate of the accrual period. Simulation studies can be incorporated into a clinical trial's planning phase to provide estimates for key accrual summaries including the mean and standard deviation of the accrual period.

**Conclusion:**

The accrual period of a clinical trial should be carefully considered, and the allocation of sufficient time for participant recruitment is a fundamental aspect of planning a clinical trial.

## Background

Clinical trials often plan to enroll hundreds to thousands of human subjects, and careful and regulated planning is employed to ensure that the trial's scientific objectives are fulfilled. However, the importance of the feasibility and timeliness of participant accrual is often minimized during the planning stage. This is unfortunate since the recruitment of human subjects into a clinical trial must be timely and is vital to the success of the trial [1,2]. In addition, a slowly progressing trial may expose the human subjects to ineffective treatments for a longer period of time than necessary, and as such, human subject protection may be compromised [2,3]. Therefore, a clinical trial must be planned with an adequate understanding of the potential accrual rate so that the clinical trial's scientific objectives can be evaluated in a timely manner.

The first step in estimating the clinical trial's accrual period is to estimate the accrual rate. In the context of a multi-center trial, each clinical center's rate will need to be estimated. One approach for evaluating each clinical center is to solicit information via a questionnaire. Content of the questionnaire could include measurements of the total number of cases currently or recently seen for the targeted condition, a listing of other clinical trials that might compete for the same patients, and a summary of the investigator's (or clinical center's) experience meeting prior accrual expectations. The completed questionnaires can be used to formulate an initial estimate of the accrual rate for each participating clinical center.

However, experience has shown the accrual period is longer than planned even when such tangible efforts are made to estimate the accrual rate. Carter [1] presented a methodological examination of this issue and developed a statistical model to estimate the accrual period of a clinical trial. It was argued that utilizing only the historical mean for the projected accrual period fails to account for the variation in the rate that may occur as the study progresses. In this brief report, we illustrate three approaches to estimating the accrual period of a clinical trial and offer discussion on potential best practices.

## Methods

### Unconditional approach

A common approach to estimating the accrual period in a clinical trial is to divide the sample size by the expected rate. Often, the expected rate for the clinical trial is estimated by summing the historical trends at each participating research site to create a study-wide estimate of the accrual rate. For example, if subjects are to be recruited from 5 sites at the expected rate of 5 participants per month per site, then one could estimate that it would take 8 months to enroll 200 participants using the combined rate of 25 participants per month. However, since the rate of 25 participants per month is assumed to be fixed in value, it is best to consider that "on average" it may take 8 months to enroll 200 participants. The obvious limitation of this approach is that there is no mechanism to account for known sources of variation in the rate. For example, if the 5 sites were to begin enrolling at different times, then a study-wide estimate of 25 participants per month is no longer tenable. The following approach directly addresses this limitation.

### Conditional rates

A more accurate estimate of the accrual period is possible when additional prior information is incorporated into the model. Two important sources of variation in the overall accrual period are the rates at which individual sites accrue and the length of time in which sites actively recruit participants. For example, suppose that a large research institution (Site A) may be capable of recruiting 15 participants per month and that four additional sites are only capable of recruiting a combined 10 participants per month. Then, the point in time in which the Site A begins enrollment will greatly affect the trial's overall accrual rate. Moreover, the timing of Site A's initiation and subsequent accrual should be critically evaluated by the sponsor.

For example, if it is known in advance that Site A will take 4 to 8 weeks longer to initiate recruitment than the other four sites, an estimate of 8 months to enroll 200 participants would seem overly optimistic. The sponsor might be interested in determining the effect of including Site A on key trial markers (e.g., feasibility, cost, and total clinical trial duration) by allowing for up to an 8-week delay in Site A's initiation. However, the calculation of a more refined accrual period, which would drive many of the cost estimates, would require the adjustment for time dependent changes in the overall accrual rate. Fortunately, computational tools such as a spreadsheet aid in the necessary calculations.

### Poisson process

The methodology presented by Carter [1] allows for a more sophisticated model that can incorporate a variety of sources of variations. In this paper, the exploration of mean accrual time will be examined. As the two previous approaches illustrate, the mean number of participants per month per site was assumed to be fixed, yet the overall accrual time varied. The source of variation introduced was a delay in the initiation of the protocol at a site. There is another source of variation. Just as is well established in economics, historical trends may not adequately reflect future trends especially when short periods of time are considered.

One approach to incorporating variation into mean number of participants per month is to assume that participants arrive into the study according to a known probability distribution. Carter [1] proposed the Poisson distribution since it has widespread applications that often include the statistical modeling of the number of arrivals or observations [4,5]. Essentially, this method simulates the accrual into a trial using a random number generator. At the end of each simulation iteration, the number of days (or months) required for the program to reach the sample size is recorded. When the simulations are repeated many times, an empirical distribution of the accrual period is obtained. Using this empirical distribution, one can answer questions related to the probability of completing accrual within a specified time as well as observe how much variance in accrual time can be expected given the model assumptions. This probabilistic approach is most beneficial when a finite amount of time has been granted for the clinical trial. Thus by conditioning on the predetermined time, one could estimate the number of clinical centers and the rate per month needed to ensure with high probability, such as 80%, that the clinical trial will complete enrollment before the allocated time expires.

## Results

To illustrate these three approaches, a multi-center protocol undergoing development is presented. The primary objective of the study is to measure the effectiveness of a selective serotonin reuptake inhibitor (SSRI) in the treatment of depression in a patient population with comorbid substance dependence. A sample size of 360 participants, enrolled from 10–12 research sites, has been estimated to test the primary hypothesis with 90% power. While the protocol is still being finalized, an initial estimate of the accrual period is of practical interest to the protocol investigators.

The final selection of the research sites has not been made, but to be considered for the trial, each site must demonstrate a patient population that would support enrolling two participants per month in the trial. In addition, it is anticipated that the protocol will be rolled out initially at one to two sites with the remaining sites coming online three to eight months later. Table 1 presents the results of the presented models as well as a variation of the Poisson process model that allows for fluctuations in assumed accrual rate over time. For simplicity, each site's accrual rate is assumed to equal two participants per month and ten sites (instead of up to 12) have been chosen so that a conservative estimate of the accrual period is obtained. Using these assumptions and the unconditional approach, it is estimated that 18 months would be required (360 / (2 * 10)) to accrue the participants. However, this calculation does not account for the delayed start of the majority of the sites.

**Table 1:** Estimated accrual periods for a 360-participant, 10-center clinical trial.

	Unconditional Approach^*a*^	Conditional Rates^*b*^	Poisson Process^*c*,*d*^	Poisson Process Variable Rates^*c*,*e*^
Estimated Accrual Period					(in months)
Mean	18	23	23.2	42.6
Standard Deviation	NA	NA	1.0	8.0
Range	NA	NA	[20.1, 25.8]	[28.0, 84.0]
75th Percentile	NA	NA	24.0	46.0

^*a*^10 sites enrolling at a rate of 2 participants per month.

^*b*^For the conditional rate, the rate per month is illustrated in Figure 1.

^*c*^Based on 500 simulation iterations

^*d*^A rate of 2 participants per month with recruitment on 5 days a week

^*e*^A rate uniformly distributed on [0,2] with recruitment on 5 days a week

**Figure 1:**
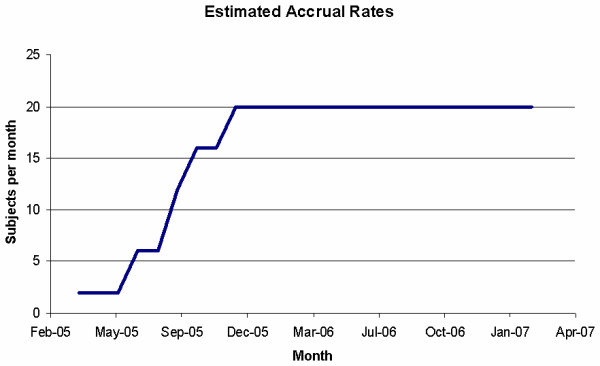
Expected accrual rate by month assuming an April 2005 start date.

To account for the delay, the conditional model could be utilized. Suppose the initial rate of accrual is two participants per month for the first two months while the protocol is active at only one site. At the start of month three, one additional site will be released for enrollment to bring the estimated accrual rate to four participants per month. Figure 1 illustrates the expected accrual as additional sites are added to the study and that full accrual potential is not reached until approximately 1/3 of the trial duration has passed. The estimate of 23 months seems much more reasonable given the staggered initiation process; however, additional models could be implemented to examine the effects of fluctuations in the accrual rates. Two separate Poisson models are presented in Table 1. Both models use the initiation pattern of the conditional model; however, the daily rate, which is the monthly rate divided by the standard number of days in the month, has been adjusted to reflect anticipated enrollment dynamics. Specifically, while the clinical sites treat potential participants every day of the week, research staff are expected to be available only five days per week. Thus, the estimated rate per month is discounted effectively by 5/7 to reflect this anticipated pattern. The second Poisson model further adjusts the estimate of 2 participants per month downward to be uniformly distributed between zero and two. Such an adjustment might be necessary to account for the difference in the number of participants eligible for the trial and the number that will actually consent to participate in the research. In addition, this reduction in the overall rate also adjusts the rate downward for other competing demands that could affect the participant accrual rate but would be difficult to quantify directly. As Table 1 illustrates, a dramatic difference in the expected accrual period is obtained if the rate is allowed to fluctuate uniformly on the interval 0 to 2; however, the conditional approach and the fixed-rate Poisson model provide similar results with the exception that the Poisson-based approach provides additional summary statistics that may be of interest during the planning phase.

## Discussion

The estimation of the accrual period of a clinical trial has dramatic practical, scientific and economic consequences and warrants greater attention in practice. As our example illustrates, the unconditional approach, while simple to implement, can yield results not consistent with trial assumptions, and may endanger the successful completion of the trial. It is acknowledged that the most complicated aspect pertaining to the estimation of accrual periods is the determination of the expected rate. As our example illustrates, sometimes dramatically different results occur with what are apparently minor modifications to model assumptions. When the potential impact of a poorly accruing clinical trial is considered, spending additional time examining the effects of model assumptions is well justified. In the end, accounting for variation and planning are essential components of good clinical research.

For the research study under consideration in this example, an estimate of 24 months to complete study recruitment seems reasonable. An accrual rate of at least two participants per month is the minimum requirement, so the expected rate after adjustment for screening to enrollment dropout percentages should still yield an accrual rate of approximately two per month. However, a more refined estimate of the accrual period can be obtained once final selection of the research sites has been made and all historical information has been analyzed. These models can also be applied after study enrollment has begun, and in the case of the Poisson model, estimating the probability of meeting the established accrual deadline has several practical implications. Although the methods have been presented in the context of a multi-center clinical trial, they are valid in the planning of a single-center clinical trial.

To facilitate implementation of the models in practice, a spreadsheet template for the calculation of the conditional model and SAS programs for the Poisson process are posted on the first author's website . Each may be freely downloaded and implemented with very little training. The posted conditional model allows for individual sites to enroll at site-specific rates as well as provides means to quickly adjust model assumptions so that sensitivity analyses can be performed. A more detailed discussion of the Poisson method is available elsewhere [1]. In summary, the conditional and Poisson accrual estimation methods may be useful to researchers designing a complex, multi-center clinical trial.

## Conclusion

The accrual period of a clinical trial should be carefully considered, and the allocation of sufficient time for participant recruitment is a fundamental aspect of planning a clinical trial. For most multi-center clinical trials, the conditional approach should be implemented. Simulation studies using the Poisson model are recommended if there is uncertainty in the accrual rate or if the accrual rate is expected to change over time.

## Competing interests

The author(s) declare that they have no competing interests.

## Authors' contributions

RC conceptualized the three accrual models and performed the analyses. SS and KB provided critical input into the model assumptions as well as the conceptualization of the referenced research project. All authors participated in the writing and editing of the manuscript.

## Pre-publication history

The pre-publication history for this paper can be accessed here:


